# Knowledge Assessment of COVID-19 Symptoms: Gender Differences and Communication Routes for the Generation Z Cohort

**DOI:** 10.3390/ijerph17196964

**Published:** 2020-09-23

**Authors:** Irene (Eirini) Kamenidou, Aikaterini Stavrianea, Spyridon Mamalis, Ifigeneia Mylona

**Affiliations:** 1 Department of Management Science and Technology, International Hellenic University, Kavala Campus, 654 04 Agios Loukas Kavala, Greece; mamalis@mst.ihu.gr (S.M.); imylona@mst.ihu.gr (I.M.); 2 Department of Communication and Media Studies, National and Kapodistrian University of Athens, 105 62 Athens, Greece; aikstavria@media.uoa.gr

**Keywords:** generation Z cohort, COVID-19 symptoms, knowledge assessment, gender differences, marketing communication

## Abstract

This paper explores the generation Z (Gen Z) cohort’s self-assessed knowledge regarding the coronavirus disease 2019 (COVID-19) symptoms as well as their interest in acquiring information and learning more about the transmission and spread of the severe acute respiratory syndrome coronavirus 2 (SARS-COV-2 virus) and the COVID-19 symptoms. Additionally, it investigates gender differences in self-assessed knowledge of COVID-19 symptoms. Field research employing a nonprobability sampling method with an online questionnaire resulted in collecting 762 valid questionnaires. Data analysis included descriptive statistics, factor and reliability analysis, and the independent sample *t*-test. Results reveal that overall symptom knowledge was assessed higher than the self-assessed knowledge of the 13 specific symptoms. No gender differences were detected regarding self-assessed knowledge of the following COVID-19 symptoms: cough, dyspnea, anorexia, productive cough with expectoration (phlegm), headache, and diarrhea. On the other hand, for self-assessed overall knowledge of COVID-19 symptoms, as well as self-assessed knowledge of COVID-19 symptoms related to fever and fatigue, myalgia (muscle pain), pharyngodynia, nausea–vomitus, hemoptysis, and abdominal pain, the *t*-tests conducted showed that there are statistical differences in knowledge assessment between male and female subjects. Based on the outcomes, the paper provides marketing communication practices targeting this young generation cohort to raise awareness so that Gen Z’ers may react effectively if these symptoms are observed and, thus, request medical assistance.

## 1. Introduction

The coronavirus disease 2019 (COVID-19) has affected the global community resulting in 31,343,430 confirmed cases globally and 965,250 deaths as of September 22, 2020 [1]. The three most-infected countries globally are USA, India, and Brazil, with 6,858,010; 5,562,663 and 4,558,040 confirmed cases, respectively. These cases of COVID-19 led to 199,886; 137,272; and 88,935 deaths in USA, Brazil, and India, respectively. COVID-19 disease originates from the severe acute respiratory syndrome coronavirus 2 (SARS-CoV-2). It was initially recognized in Wuhan, Hubei province, China, in December 2019 [2], while in January 2020, it was already declared by the World Health Organization (WHO) as a public health emergency of international concern [3]. Two months later (i.e., in March 2020), it had already developed to a pandemic.

The disease is characterized by various symptoms, appearing during a range from 2 to 14 days after exposure. Though 97.5% of patients develop symptoms within approximately 11–12 days after infection [4,5,6,7]. The virus seems to spread through droplet transmission [4] as well as fecal-oral transmission [8,9,10], with symptoms ranging from very mild to very severe [4], while certain people infected may not experience any symptoms at all [11].

The most common symptoms include fever, cough, dyspnea, myalgia, and fatigue [2,5,12,13,14,15]. Other symptoms are anorexia, nausea, and diarrhea [2,4,5,13,14,16]. Additionally, it is reported that just before or soon after the symptom onset, people that have been affected by the virus have high nasopharyngeal viral levels that fall over the course of approximately one week [17]. One of the fundamental problems in combating the spread of the SARS-CoV-2 virus and, thus, the COVID-19 disease is that pre-symptomatic and asymptomatic people are infectious. A 40% to 50% of the cases are attributed to transmission from asymptomatic or pre-symptomatic individuals [18].

Age is considered a risk factor of COVID-19, especially for individuals above 65 years old. At the same time, cardiovascular problems, chronic lung disease, hypertension, diabetes, and obesity are also connected with a high risk of being infected by the SARS-CoV-2 virus [4,6,13,19]. On the other hand, while young people are not classified at direct risk of COVID-19 mortality, they are considered as virus transmission-risk associated, in the sense that in most cases, they are asymptomatic but are a potential spread source of the SARS-CoV-2 virus. Therefore, it becomes of crucial significance to know whether young people are informed of the symptoms of COVID-19 since humanity is faced with this long-lasting pandemic. These young individuals might be called to deal with a severe health condition due to the SARS-CoV-2 virus regarding friends, relatives, or even themselves. It is imperative to ensure that young people are fully conscious of the disease’s symptoms, in case they spot them in relatives or friends. If this situation appears, they will be able to support them before the problem is beyond their control, while they might be the ones who have infected them in the first place.

Since the COVID-19 disease is a continuing health crisis, previously published research regarding SARS-CoV-2 virus and COVID-19 disease is significant to better comprehend the spread of the virus and prevent the COVID-19 disease. Gaining insights from the young people about their knowledge regarding the symptoms of COVID-19 is equally crucial since their knowledge may project their future behavior if they face a health issue in their environment due to the virus. Thus, symptom knowledge is critical in order to adopt a quick response and prevent the spread or unpleasant developments or outcomes of the disease.

Therefore, the subsequent research questions arose from the related literature and the significance of peoples’ knowledge regarding COVID-19 symptoms:

RQ1: Do young people believe that they are familiar with the symptoms of COVID-19?
RQ2: Do young people want to learn more about the transmission patterns of the virus, its spread, and its symptoms?
RQ3: Do male and female subjects express the same level of self-assessed knowledge regarding the symptoms of COVID-19?
RQ4: What marketing communication techniques should be implemented in order to increase young people’s awareness of COVID-19 symptoms?

For this research, data were gathered from Greece. Greece had taken proactive measures in an early stage, thus managing to have a rather small number of COVID-19 related confirmed cases and deaths [20].

Specifically, data for this research is drawn from the Greek generation Z cohort. The generation Z cohort comprises of individuals born between 1995–2009 [21,22]. This research draws a sample from the adult citizens of the Greek generation Z cohort, being today (i.e., in 2020; year in which the research was conducted) 18–25 years old (thus born 1995–2002). Earlier studies have evidenced that consumer behavior depends on age [23,24], and that different age groups demonstrate distinct behavior [25,26], with scientists indicating the significance of using generational cohorts in consumer behavior studies [27,28,29]. It must be highlighted that generations and generational cohorts are two different concepts. Specifically, generations are defined by a time frame of 20–25 years. Generational cohorts refer to individuals born within a specific time frame, specific area, and have lived the same life-changing events when they are 17–23 years of age. It is believed that common experiences and events formulate shared values and the establishment of similar behaviors that define each cohort [22,30,31,32]. The generation Z members are digitally savvy, and because of their relationship with technology, “Gen Z’ers” are also known as the “Digital natives,” “Homo Zappiens,” the “Google generation,” the “Net Generation,” or the “Dot-com kids” [33,34,35]. The members of this generation still experience circumstances that shape their values system and personality. The members of this generational cohort constitute a pole of great academic interest, since they are the youngest adult consumers who will make their own families in the near future [36]. It is generally recognized that a more in-depth knowledge of Gen Z’ers can lead to a better understanding of the significance of the different generations in contemporary society [33,34,35].

Gen Z’ers have been faced with major political, economic, and social changes. The Greek generation Z members have experienced and are still enduring the harsh effects of the financial crisis that the country faces since 2010 [37,38]. They have undergone capital controls, the rise of the extreme right political party Golden Dawn (i.e., Chrisi Avgi), a left government, and large numbers of young and educated Greeks migrating to other countries in pursuit of better employment conditions and a more promising future. This year, they have faced the COVID-19 pandemic and the country’s lockdown.

Insights from this youngest adult cohort is exceptionally important because they might reflect the behavior of the age group that is on the one hand asymptomatic in almost all cases, whereas, on the other hand, they may be infectious without knowing it—and thus a “moving threat” for the older and vulnerable population. Lastly, because in their majority they live together with parents and grandparents (due to the economic crisis that Greece has been facing for the last 11 years), they must be knowledgeable of the symptoms of COVID-19, in case they encounter them in family members. Therefore, they should be able to recognize them in order to request quickly medical help.

From the abovementioned research questions, the overarching aim of this research is to examine the Greek generation Z cohort’s self-assessed knowledge of the COVID-19 symptoms (overall and for 13 specific symptoms, answering RQ1). Furthermore, its objectives are to (1) explore if Gen Z’ers are willing to learn more about COVID-19, specifically regarding the transmission and spread of the virus, as well as the disease’s symptoms (answering RQ2); (2) examine whether males and females Gen Z’ers express the same levels of self-assessed knowledge regarding the signs of COVID-19 (answering RQ3); and lastly, (3) recommend marketing communication techniques targeting to raise awareness and increase understanding of COVID-19 symptoms for this specific generational cohort (answering RQ4).

While there is a rapid research boost and academic articles published concerning COVID-19, this research adds to the prior academic work in the subsequent ways:

1. It provides insights from a generational cohort that has not been studied (to our knowledge) regarding the COVID-19 disease. While one article has been found that deals with generational differences, it focuses solely on perceptions of food health-risk and attitudes toward organic food and game meat during the COVID-19 crisis in China [39]. Though, that article does not deal with COVID-19 symptoms and deals with generations and not generational cohorts.
2. It studies self-assessed knowledge of COVID-19 symptoms, which is at present an understudied issue (42 academic papers exist to our knowledge). While COVID-19 is subject to extensive ongoing research, areas of main focus as regards individuals’ behavior mainly focus on the psychological impact of COVID-19, impact on work and telework, and protective measures that citizens comply with, e.g., [20,40,41,42,43,44,45,46].
3. It provides an in-depth insight of research referring to young people’s self-assessed knowledge regarding the symptoms of COVID-19, an understudied topic too. Specifically, only three peer-reviewed academic research papers were found that examine young peoples’ knowledge of COVID-19 [47,48,49]. In these three papers, the majority used a sample of university students, drawn from health care and non-healthcare areas of study. The present research’s main criteria for inclusion were not to be employed in the health-care domain or be students in any healthcare school or department.
4. It examines young peoples’ interest in learning more about COVID-19 symptoms as well as SARS-CoV-2 virus transmission and spread, two issues that to our knowledge have not been dealt with.
5. It offers a detailed reflection of the youngest adult generation cohort’s behavior in crises situations, since information and risk knowledge can influence behavior [50]
6. It explores gender differences of individuals of the generation Z cohort regarding self-assessed knowledge connected to COVID-19 symptoms, which to our knowledge has not been studied yet. While gender differences regarding various issues connected to COVID-19 has been investigated, no research has been found to be focused on gender differences from the specific generation cohort and COVID-19 self-assessed symptom knowledge.

## 2. Materials and Methods

### 2.1. Research Design

Within the existing theoretical framework, this study’s data were gathered employing a questionnaire created explicitly for this reason based on prior researches, e.g., [2,4,5,12,13,14,15,16].

A pilot test (*n* = 28) led to modifications concerning syntax and grammar errors, simultaneously ensuring face validity [20]. A combination of non-probability sampling methods was used (criteria, convenience, and snowball) utilizing online platforms. The main criteria that this research posed was that participants should be adult members of the Greek generation Z cohort (Gen Z’ers) and not be in any way connected with the healthcare sector (e.g., workers or medical/healthcare students). The questionnaire used was developed with Google Forms, and its link was distributed mainly through social media (Facebook) and email in the circumstance that acquaintances did not have a Facebook account (convenience sampling). Lastly, participants were encouraged to call for participation acquaintances, friends or relatives that fell into the above criteria (i.e., adult Gen Z’ers and not connected in any way with the healthcare sector), utilizing in this manner the snowball sampling procedure. The data collection process applied an online questionnaire which remained active from 1 March to 13 May 2020. The initial number of the gathered data was 824 questionnaires, although 62 were discarded due to various problems or inconsistencies (e.g., did not give consent for using data, had conflicts in answers). Thus, the final sample, consisted of 762 valid questionnaires, producing a response rate of 92.5%. This sample size was considered adequate for the statistical analysis employed [51].

Ethical approval: “There are no ethical issues involved in the processing of the questionnaire data used in the study. The necessary consents have been obtained by the persons involved, and the anonymity of the participants has been secured. All procedures performed in studies involving human participants were in accordance with the ethical standards of the International Hellenic’s University research committee and with the 1964 Helsinki declaration and its later amendments or comparable ethical standards”. Permission was obtained with the No. 2/20.1.2020 decision of the Coordinating Committee.

### 2.2. Measures

Self-assessed knowledge of COVID-19 symptoms included two questions. The first question was presented as “How would you rate your level of knowledge of the COVID-19 symptoms?” This question was rated on a seven-point Likert-type scale (1 = I have absolute ignorance (I do not have any knowledge at all), 2 = I am quite ignorant/unaware, 3 = I am somewhat ignorant/unaware, 4 = I have neither ignorance/nor knowledge (neutral), 5 = I have some knowledge, 6 = I have quite a lot of knowledge, 7 = I have absolute knowledge). This question is considered as the “overall” knowledge assessment regarding COVID-19 symptoms. Then, participants were requested to assess their knowledge on thirteen specific symptoms of the COVID-19 disease adopted from previous literature. This 13-multi-item question was addressed as follows: “How would you rate your level of knowledge regarding the following symptoms of the COVID-19 disease?” The scale implemented was identical with the scale of overall knowledge.

The data analysis covers descriptive statistics, i.e., frequencies, percentages (%) and mean scores (addressing RQ1 and RQ2) and *t*-tests (answering RQ3 of the study). The significance level in the hypothesis testing procedures (*t*-tests) was preset at a= 0.05 (*p* < 0.05).

Additionally, the reliability and validity of the multi-item question regarding COVID-19 symptoms were checked. The reliability of the scale was computed with Cronbach alpha to confirm the internal consistency of the scale, producing Cronbach α = 0.919, which is considered acceptable [52]. Content validity was confirmed by adopting the items from the abovementioned peer-reviewed academic published papers. The pilot test also confirmed face validity by introducing an additional question regarding readability and understanding of questions and the questionnaire in its totality, as well as suggestions for better comprehension and use [34,53]. Moreover, convergent and discriminant validity was tested using, in both cases, Fornell and Lacker’s [54] equation/criteria. Referring to convergent validity, it was assessed by average variance extracted (AVE), and composite reliability (CR) both measured for the multi-item question, i.e., two constructs (produced from factor analysis with varimax rotation). The AVE result for construct No.1 was 0.583 and for construct No.2 it was 0.585, both >0.5, and as such, convergent validity of the constructs is acceptable [54]. Likewise, Fornell and Lacker [54] ascertain that discriminant validity is recognized when the correlations among the constructs are lower than the square root of the AVE. Analysis revealed that discriminant validity is established.

## 3. Results

### 3.1. Sample Profile

Male subjects were under-represented (Table 1). Regarding participants’ age, the younger Gen Z’ers (18 and 19 years old) are overrepresented, while the remaining ones tend to coincide in percentages. Summarizing their profile, they are single, with secondary education, dependent on others (students in their majority), residing in urban areas, and with a personal net income of ≤350.00€ per month, by average.

### 3.2. Self-Reported Knowledge Assessment-RQ1/Main Aim of the Study

Gen Z’ers were invited to report their self-assessed knowledge regarding the COVID-19 symptoms (RQ1/main aim of the study), rated on a 7-point Likert type scale, (1 = I have absolute ignorance, up to 7 = I have absolute knowledge). Table 2 presents participants’ answers in percentages and mean scores (MS) regarding self-assessed overall knowledge of the COVID-19 symptoms. Results show that Gen Z’ers estimate that they possess a wealth of (overall) knowledge (MS > 5.50) of COVID-19 symptoms.

Subsequently, participants were requested to report their level of self-assessed knowledge regarding 13 specific symptoms of COVID-19, presented on the same 7-point Likert type scale (Table 3); each number in the scale of 1–7 in the first row resembles the numbers of the Likert-type scale, and MS correspond to mean scores. Table 3 uncovers that, generally, participants reported having some knowledge about the COVID-19 symptoms. More precisely, the symptoms for which Gen Z’ers have reported high knowledge are fever (MS = 5.9), cough (MS = 5.8), and dyspnea (MS = 5.6). In contrast, the symptoms that the Gen Z’ers assessed as having the least knowledge were the following: hemoptysis (MS = 3.3), pharyngodynia (MS = 3.7), abdominal pain and anorexia (both with MS = 3.8).

### 3.3. Willingness to Learn about COVID-19 Symptoms-RQ2/Objective N.1

Gen Z’ers were asked if they would be interested in obtaining more information about the SARS-COV-2 virus and especially with respect to its transmission and spread, as well as in relation to the COVID-19 symptoms (RQ2/objective N.1 of the study). Table 4 reveals that overall, there is an interest from Gen Z’ers to be more informed about these topics, with the information regarding symptoms attracting the highest interest response rate and the information about the virus spread exhibiting the lowest.

### 3.4. Gender Differences-RQ3/Objective N.2

Gender differences regarding self-assessed knowledge of COVID-19 symptoms were examined (answering RQ3/Objective N.2). *T*-tests for independent samples (SPSS ver. 25) for overall knowledge and separately for each symptom as well, i.e., 13 symptoms in total are implemented (Table 5 and Table 6).

Table 5 summarizes the group statistics for gender and self-assessed knowledge of COVID-19 symptoms. Among the 14 cases tested (13 specific symptoms and overall knowledge assessment), female subjects assessed knowledge higher than male subjects in only five categories, namely, for overall knowledge and for four symptoms: fever, cough, fatigue, and dyspnea.

Table 6 presents the independent sample *t*-test for self-assessed overall knowledge of COVID-19 symptoms and self-assessed knowledge for 13 specific symptoms, i.e., 14 cases in total (with even variances being assumed). The *t*-tests unveiled that for eight out of fourteen cases gender differences do exist.

Specifically, no gender differences were detected regarding self-assessed knowledge of the following COVID-19 symptoms: cough, dyspnea, anorexia, productive cough with expectoration (phlegm), headache, and diarrhea. On the other hand, the *t*-test showed that there are statistical differences in knowledge assessment between male and female subjects of Gen Z’ers for self-assessed overall knowledge of COVID-19 symptoms, as well as self-assessed knowledge of COVID-19 symptoms referring to fever and fatigue (F = 11.949, *p* = 0.010; F = 7.485, *p* = 0.047; F = 1.435, *p* = 0.001; respectively, for overall knowledge, fever, and fatigue). Lastly, with respect to myalgia (muscle pain), pharyngodynia, nausea–vomitus, hemoptysis, and abdominal pain, results also indicate that gender differences exist too (F = 0.980, *p* = 0.023; F = 0.058, *p* = 0.032; F = 1.978, *p* = 0.025; and F = 2.541, *p* = 0.042, respectively).

## 4. Discussion

### 4.1. Self-Assessed Knowledge of COVID-19 Symptoms (RQ1-Aim of Study)

In relation to the RQ1/aim of the study, and by observing Table 2 and Table 3, it is evident that Gen Z’ers consider having adequate (overall) knowledge of the symptoms of COVID-19 (MS = 5.62 > 5.50). Additionally, the MS of the self-assessed knowledge for nine out of thirteen symptoms are <5.00, while the overall MS of the 13 symptoms is 4.58 (<5.00). This outcome reveals that Gen Z’ers have overestimated their overall knowledge of the COVID-19 symptoms and that the symptoms they are informed about are the ones mostly communicated by the government and the media. More precisely, concerning anorexia, abdominal pain, pharyngodynia, and hemoptysis, about half of the sample has limited knowledge of them being COVID-19 symptoms. This implies that, if the Gen Z’ers came across the specific symptoms rated with MS < 4.00, they would probably not be able to identify that they are connected with COVID-19, and they would possibly not pay the required attention.

These research findings can be considered partially in line with previous related research. Specifically, Zhong et al. [47] examined 6910 participants from China regarding knowledge of COVID-19 (13 items), where one of the items referred to symptoms (“The main clinical symptoms of COVID-19 are fever, fatigue, dry cough, and myalgia”), on a true-false-do not know scale. They found that 96.4% of the sample knew that these are the main symptoms of COVID-19. Alzoubi et al. [48] examined 592 university students’ (medical and non-medical) knowledge of COVID-19 symptoms. Six items were included in the questionnaire on symptom knowledge (fever, cough, difficulty in breathing, headache, vomiting, and diarrhea). Results revealed that for the most common symptoms of COVID-19 (fever, cough, difficulty in breathing), the knowledge rate was >90%, while the rest had a rate of 61–75%, the least being “Diarrhea is not a common symptom of COVID-19”.

Escalera-Antezana et al. [49] in their cross-sectional study researched knowledge of COVID-19 symptoms (amongst others) with a one question-item. The research was undertaken in Colombia and Bolivia, employing 1165 healthcare students and workers. The question was an open-ended question presented as “Regard symptoms (of COVID-19), most patients present with…?” They found that knowledge about symptoms was higher in Bolivia than Colombia preceding the intervention (relevant training seminars). Following the intervention (that is, after they completed relevant training seminars), no significant differences were attested comparing before and after for Bolivian participants, though, for Colombian participants, there was a significant increase in their knowledge of COVID-19 symptoms.

The results that the current study yielded are partially in line with those of the above researches since this research explored the self-assessed level of overall and specific-related symptoms of COVID-19 (deepened into 13 specific symptoms). It revealed that Greek Gen Z’ers are knowledgeable of the most common symptoms (fever, cough, dyspnea, and fatigue) as revealed in the afore-mentioned studies, while the symptoms that are not so frequently come across of, i.e., anorexia, abdominal pain, pharyngodynia, and hemoptysis, were not rated in the abovementioned studies.

### 4.2. Interest in Obtaining Information Regarding COVID-19 (RQ2-Objective N.1)

Commenting on RQ3/objective N.1, this study (Table 4) uncovered that Gen Z’ers are keen to learn more about SARS-COV-2 and COVID-19, with the vast majority concerned about the symptoms of COVID-19 rather than the transmission or spread of the SARS-COV-2 virus. This finding suggests that Gen Z’ers are interested in solving the problem rather than preventing it from happening. It might also mean that they are not interested in taking proactive measures or isolating themselves for their protection and the protection of others. Additionally, this outcome could be due to the lack of fear that COVID-19 could happen to them, since young people are in the overwhelming majority asymptomatic, while they undergo the disease rather painlessly. Lastly, it could just be a lack of interest in the whole issue since the mortality and symptomatic morbidity rate is exceptionally low in their age range. The results of this study cannot be compared to other studies since no previous research has dealt with this subject, though it could be generally be associated with risky behavior. Risk-taking can be defined as the engagement in behaviors that have the possibility of compromising an individual’s health and of the people around him/her [55]. Prior research has explored the impact of age on risk-taking propensity [56]. Research has shown similarities—across nations—in a heightened risk-taking propensity during adolescence, which can be increased during the second decade of a person’s life [57,58]. Furthermore, Duell et al. [58] empirically supported in a cross-national study that age has an impact on real-life risk-taking, which heightens during the mid-twenties. Other researchers have also found that health risk behaviors augment in the 20s [59,60,61,62].

### 4.3. Gender Differences (RQ3-Objective N.2)

Emphasizing on the RQ3/Objective N.2, gender differences were detected regarding self-assessed overall knowledge of COVID-19 symptoms and self-assessed knowledge of fever and fatigue as COVID-19 symptoms. In these cases, women reported higher knowledge compared to men (Table 5: means). Lastly, with respect to the symptoms myalgia (muscle pain), pharyngodynia, nausea–vomitus, hemoptysis, and abdominal pain, results also revealed gender differences, with male subjects reporting higher self-assessed knowledge of these being symptoms of COVID-19 in comparison to female subjects (Table 5: means). These results cannot be directly compared to the results of previous academic studies on the grounds that authors identified no prior study that encompassed gender differences of COVID-19 symptom knowledge.

Prior research demonstrates that, when examining young adults, females have higher risk perceptions and engage less in health risk behaviors than males [58,60,63,64]. In COVID-19 related research, Cvetkovic et al. [65] supported that women believe more than men in that this disease is contagious. In addition, females felt more vulnerable than males to the disease [66]. Furthermore, Cutler et al. [67] reported that U.S. males are less likely to know how COVID-19 is spread or the exact symptoms of the disease, while Karijo et al. [68] supported that females were more likely to name correctly the symptoms of the coronavirus than males.

### 4.4. Marketing Communication Routes Targeting Male and Females (RQ4/Objective N.3)

Individuals who belong to this generation are technology, social media, and smartphones savvy. They are surrounded by mobile devices, while social media have always been an integral part of their lives [33,69]. On the contrary, they cannot be easily reached through traditional media such as television [69]. Gen Z’ers are considered traditional, infused with family values, and they tend to undertake their responsibilities [33]. They desire to be taken seriously, they like sharing information, and they question things valuing trustworthiness. They also strive for rich experiences and intense pleasure [70]. According to a research involving twenty countries with young people aged 15–21 years old, which was conducted in 2016 by the Varkey Foundation, Gen Z’ers appreciate technology, and their values are influenced by their parents. Interestingly, though, they are pessimistic about the way the world is devoting [71].

Proactive communications play an integral role in a public health emergency and crisis and can become one of the most significant interventions in order to provide actual information [20]. As shown from the study’s results, younger citizens should be better informed about COVID-19 and the symptoms of the disease. Digital media can be employed to ensure that the targeted audience obtains regular and up-to-date information on the disease, through e.g., websites and social media. The digital environment, especially social media, is important for disseminating information and shaping the behavior of young adults [72]. Prior research has confirmed that platforms such as Facebook, Twitter, YouTube, and LinkedIn act as forums that connect users and facilitate the creation of online communities where opinions can be exchanged [72]. Social media services acted as a source of information and played an important role in health issues and crises such Mers-CoV or Zika virus [73], and especially for Gen Z’ers, social media constitute a great part of their daily routine. The sharing of stories (also targeted to the different genders), photos, and videos that demonstrate the key messages can further reinforce communication goals.

A digital media platform that offers great opportunities for targeting younger adults is YouTube with over two billion users, also combining audio and visual elements. It is one of the most preferred media for acquiring information about COVID-19 [74]. Though, prior research has shown that more than 25% of the most viewed videos on YouTube regarding COVID-19 contained misleading information (e.g., the disease only affects older people), and videos from reputable sources are under-represented [75]. Therefore, it is essential that trustworthy sources use the medium in a twofold way. Firstly, in order to inform young adults via effective strategic communication plans and, secondly, as with other social media to become a tool that authorities can use in order to listen to the opinions and beliefs of these categories of citizens. Maintaining a two-way communication is essential to comprehend and answer the concerns of Gen Z’ers and, also, to communicate the appropriate messages and information that will enable them to overcome any barriers in following precautionary measures.

Proper content and targeting and the use of various emotional tones (e.g., humor) can increase viewership and engagement of female and male Gen Z’ers. Furthermore, videos with the endorsement of celebrities and YouTubers that appeal to younger adults may extend the reach and reinforce the impact of the videos. Partnership with entertainment producers can enable the creation of material that disseminates information from official sources and at the same time has the potential to be viral amongst young adults. Individuals tend to be interested in and pay attention to news that affect them directly and personally [76]. In that context, in order to increase attention, discussion and sharing of information, youngsters need to feel that they may be impacted by COVID-19. Because of their age, young adults usually feel less vulnerable to the disease and, therefore, disregard related information and recommendations for protection measurements [77]. Therefore, communication that addresses the members of the generation Z cohort should highlight the advantages and importance of being informed about the disease and the symptoms and follow proactive measures, not only for protecting themselves but also for protecting their close ones, their society, and additionally gain in that way their peer group’s approval. Therefore, it is important to encourage discussions between members of this age group through various social media groups and also promote peer-to-peer communication.

Additionally, through these platforms, the concerns, questions, and misleading information should be closely followed to give factual answers. Within that framework, public communication should also be aligned with efforts for community engagement. Individuals who are influencers to these groups of adults as well as networks, such as various youth associations and women’s groups, could reinforce community engagement and act as mobilizers to target Gen Z’ers according to gender and interests. Furthermore, the strategic employment of social media with the use of hashtags and motives to share relevant information with friends and acquaintances can reinforce the adaptation of communication messages by young adults.

### 4.5. Limitations and Directions for Future Research

This research is characterized by certain limitations, although an attempt was made to regulate them. Firstly, a non-probability sampling method was applied, resulting in a lack of generalizability of the results. Secondly, due to economic and time constraints, the paper emphasized only on one country, i.e., Greece, and on a single generation cohort, the Gen Z’ers, resulting in a small sample compared to other studies exploring behavioral patterns towards COVID-19. Lastly, another limitation is that other symptoms that are connected to COVID-19 could have been also examined but the ones reported in this study are the most referred to in the pertinent literature.

Unquestionably, these limitations provide fertile ground for future research; among potential future directions, scholars may consider the expansion to more generational cohorts (generation X, generation Y, Baby Boomers, and GI generation), as well as using a probability sampling frame and a larger sample. Finally, it would be thought-provoking to apply this study to other countries’ generation Z cohorts in order to measure self-assessed knowledge of COVID-19 symptoms and compare results.

Even though this study has the above-mentioned limitations, its contribution to the academic research is significant, as it offers requisite input regarding a generational cohort that has not been studied under the prism of COVID-19, while it also provides country-focused managerial suggestions in order to raise symptom awareness and public knowledge.

## 5. Conclusions

The current research offered novel insights into the Greek generation Z cohort regarding self-assessed knowledge of COVID-19 symptoms. The present study contributes significant findings that can assist researchers and government officials in developing a better understanding of the generation Z cohort and their gender differences associated with the self-assessed level of knowledge regarding the COVID-19 disease symptoms. Firstly, it has become evident that Greek Gen Z’ers are knowledgeable of the common—most frequent symptoms of COVID-19. Additionally, based on the mean scores of the self-assessed overall and the thirteen specific symptoms of COVID-19, it seems that subjects have overrated their self-assessed knowledge regarding the specific disease’s symptoms. Precisely, when enquired about their overall knowledge about the disease’s symptoms, they rated themselves highly, but when they were asked to assess their knowledge on specific symptoms related to COVID 19, they were not familiar with them to a confident degree. Thirdly, it has been demonstrated that gender differences do exist regarding symptom self-assessed knowledge of COVID-19. Based on these gender differences, better targeted communication routes should be implemented in the direction of raising Gen Z’ers’ awareness and knowledge. Some main communication routes are the digital paths, since this cohort is technologically savvy, and in general, they abstain from following traditional information routes. As Kaplan [69] (p. 9) reports (they) “don’t read newspapers (which is why newspapers will likely disappear in the near future), don’t watch regular TV (at least not without passing it through a TiVo), and are surrounded by their personal mobile devices most of the time (which makes it nearly impossible to reach them through billboards or radio). But the fact that social media have always been part of their lives—Facebook was founded in 2004, YouTube in 2005, and Twitter in 2006—makes them perfect candidates for mobile social media applications.”

## Figures and Tables

**Table 1:** Participants’ profile.

Sample Characteristics	Frequencies	Percentages (%)
Gender		
Male	315	41.3
Female	447	58.7
Age		
18–19	237	31.1
20–21	194	25.5
22–23	177	23.2
24–25	154	20.2
Marital status		
Single	734	96.9
Married/Divorced/Widowed	24	3.1
Education		
Secondary (Lyceum)	384	50.4
Postsecondary	137	18.0
Graduate/Postgraduate	241	31.6
Profession		
Employee (public-private)	127	16.7
Businessman/Businesswoman	31	4.1
Labourer	12	1.6
Student	342	44.8
Housekeeper	57	7.5
Unemployed	193	25.3
Area of residence		
Urban	462	60.9
Rural	300	39.1
Net monthly personal Income (€)		
≤350.00	466	61.2
350.01–1000.00	169	22.2
1000.01+	127	16.6

**Table 2:** Self-assessed (overall) knowledge of COVID-19 symptoms.

Statements	*n*	%
1 = I do not have any knowledge at all (absolute ignorance)	21	2.8
2 = I am quite ignorant/unaware	23	3.0
3 = I am somewhat ignorant/unaware	35	4.6
4 = I neither have ignorance/nor have knowledge (neutral)	65	8.5
5 = I have some knowledge	125	16.4
6= I have quite a lot of knowledge	223	29.3
7 = I have absolute knowledge	270	35.4
Total	762	100.0
Mean Score (MS)	5.62	

**Table 3:** Gen Z’ers self-reported knowledge of 13 specific COVID-19 symptoms (%).

Statements	1	2	3	4	5	6	7	MS
Fever	5.2	2.5	3.0	4.9	13.4	10.9	60.1	5.9
2. Cough	4.2	4.1	3.7	5.6	13.3	13.1	56.0	5.8
3. Dyspnea	5.5	3.9	4.5	6.6	17.2	13.6	48.7	5.6
4. Fatigue	6.6	5.1	7.2	12.2	17.7	14.3	36.9	5.2
5. Headache	7.9	8.0	8.1	13.5	17.5	16.4	28.6	4.9
6. Myalgia (muscle pain)	9.6	7.6	9.7	18.1	15.7	11.5	27.7	4.7
7. Productive cough with expectoration (phlegm)	11.5	7.6	8.1	15.6	15.4	13.8	28.0	4.7
8. Diarrhea	16.9	9.2	10.6	16.7	12.9	13.1	20.6	4.2
9. Nausea-Vomitus	17.3	11.5	11.4	18.9	12.9	10.0	18.0	4.0
10. Anorexia	20.9	12.1	13.3	17.2	12.2	9.2	15.2	3.8
11. Abdominal pain	22.7	11.5	13.0	13.8	13.0	8.5	17.5	3.8
12. Pharyngodynia	20.2	13.8	12.1	19.0	10.8	9.3	14.8	3.7
13. Hemoptysis	30.3	13.3	12.5	16.4	8.4	8.4	10.8	3.3

**Table 4:** Interest to obtain information on SARS-COV-2 and COVID-19.

Willingness to Learn about the…	Yes		No	
	*n*	%	*n*	%
1. Virus transmission	523	68.6	239	31.4
2. Virus spread	506	66.4	256	33.6
3. Disease symptoms	560	73.5	202	26.5

**Table 5:** Group statistics of gender and self-assessed knowledge of COVID-19 symptoms.

Symptoms	Gender	*n*	Mean	Std. Deviation	Std. Error Mean
Overall self-assessed level of knowledge	Male	315	5.286	1.6222	0.0914
	Female	447	5.566	1.3706	0.0648
Fever	Male	315	5.771	1.7806	0.1003
	Female	447	6.020	1.6450	0.0778
Cough	Male	315	5.695	1.7547	0.0989
	Female	447	5.928	1.6739	0.0792
Myalgia (muscle pain)	Male	315	4.489	1.9926	0.1123
	Female	447	4.819	1.9594	0.0927
Fatigue	Male	315	4.933	1.9283	0.1086
	Female	447	5.385	1.8104	0.0856
Dyspnea	Male	315	5.498	1.8432	0.1039
	Female	447	5.700	1.7410	0.0823
Anorexia	Male	315	3.806	2.0574	0.1159
	Female	447	3.732	2.0876	0.0987
Productive cough with expectoration (phlegm)	Male	315	4.689	2.0869	0.1176
	Female	447	4.687	2.0157	0.0953
Headache	Male	315	4.914	1.8858	0.1063
	Female	447	4.861	1.9692	0.0931
Pharyngodynia	Male	315	3.927	2.0928	0.1179
	Female	447	3.602	2.0273	0.0959
Nausea-Vomitus	Male	315	4.203	2.0024	0.1128
	Female	447	3.861	2.1077	0.0997
Diarrhea	Male	315	4.273	2.0351	0.1147
	Female	447	4.168	2.1681	0.1025
Hemoptysis	Male	315	3.549	2.0949	0.1180
	Female	447	3.083	2.0189	0.0955
Abdominal pain	Male	315	3.971	2.1181	0.1193
	Female	447	3.649	2.1698	0.1026

**Table 6:** Independent samples *t*-test between the Gen Z’ers gender and their self-assessed knowledge of the COVID-19 disease symptoms.

Overall Knowledge and Specific-Symptom Knowledge	*T*-Test for Equality of Means							
	F	t	df	Sig. (2-Tailed)	Mean Difference	Std. Error Difference	95% Confidence Interval of the Difference	
							Lower	Upper
Overall self-assessed level of knowledge	11.949	−2.575	760	0.010	−0.2803	0.1089	−0.4940	−0.0666
Fever	7.485	−1.986	760	0.047	−0.2487	0.1252	−0.4945	−0.0029
Cough	3.653	−1.856	760	0.064	−0.2332	0.1256	−0.4798	0.0134
Myalgia (muscle pain)	0.980	−2.273	760	0.023	−0.3299	0.1452	−0.6149	−0.0449
Fatigue	1.435	−3.299	760	0.001	−0.4515	0.1368	−0.7201	−0.1828
Dyspnea	3.448	−1.538	760	0.125	−0.2018	0.1312	−0.4594	0.0558
Anorexia	0.515	0.490	760	0.624	0.0748	0.1527	−0.2249	0.3745
Productive cough with expectoration (phlegm)	1.232	0.014	760	0.989	0.0021	0.1505	−0.2933	0.2975
Headache	1.495	0.372	760	0.710	0.0530	0.1424	−0.2265	0.3325
Pharyngodynia	0.058	2.151	760	0.032	0.3252	0.1512	0.0285	0.6219
Nausea-Vomitus	1.978	2.251	760	0.025	0.3419	0.1519	0.0437	0.6401
Diarrhea	4.550	0.677	760	0.499	0.1052	0.1555	−0.2001	0.4105
Hemoptysis	0.773	3.092	760	0.002	0.4664	0.1509	0.1703	0.7626
Abdominal pain	2.541	2.041	760	0.042	0.3227	0.1581	0.0124	0.6329
